# Update of Diagnosis and Targeted Therapy for ALK^+^ Inflammation Myofibroblastic Tumor

**DOI:** 10.1007/s11864-023-01144-6

**Published:** 2023-11-08

**Authors:** Qi-An Wang, Huan-Wu Chen, Ren-Chin Wu, Chiao-En Wu

**Affiliations:** 1 School of Medicine, College of Medicine, Chang Gung University, Taoyuan, Taiwan; 2 Division of Emergency and Critical Care Radiology, Department of Medical Imaging and Intervention, Chang Gung Memorial Hospital, Chang Gung University, Linkou, Taiwan; 3 Department of Pathology, Chang Gung Memorial Hospital at Linkou and Chang Gung University, Taoyuan, Taiwan; 4 Division of Hematology-Oncology, Department of Internal Medicine, Chang Gung Memorial Hospital at Linkou, Chang Gung University College of Medicine, Taoyuan, Taiwan

**Keywords:** Inflammatory myofibroblastic tumor, Anaplastic lymphoma kinase, Crizotinib, Ceritinib, Alectinib, Brigatinib, Lorlatinib

## Abstract

Inflammatory myofibroblastic tumor (IMT), characterized by intermediate malignancy and a propensity for recurrence, has presented a formidable clinical challenge in diagnosis and treatment. Its pathological characteristics may resemble other neoplasms or reactive lesions, and the treatment was limited, taking chemotherapies as the only option for those inoperable. However, discovering anaplastic lymphoma kinase (ALK) protein expression in approximately 50% of IMT cases has shed light on a new diagnostic approach and application of targeted therapies. With the previous success of combating ALK^+^ non-small-cell lung cancers with ALK tyrosine kinase inhibitors (TKIs), crizotinib, a first-generation ALK-TKI, was officially approved by the U.S. Food and Drug Administration in 2020, to treat unresectable ALK^+^ IMT. After the approval of crizotinib, other ALK-TKIs, such as ceritinib, alectinib, brigatinib, and lorlatinib, have proven their efficacy on ALK^+^ IMT with sporadic case reports. The sequential treatments of targeted therapies in may provide the insight into the choice of ALK-TKIs in different lines of treatment for unresectable ALK^+^ IMT.

**Supplementary Information:**

The online version contains supplementary material available at 10.1007/s11864-023-01144-6.

## Introduction

Inflammatory myofibroblastic tumor (IMT) is a locally aggressive mesenchymal tumor with lymphocyte infiltration [1–4], and myofibroblast spindle cell proliferation [5], that presents as a solitary lesion [6]. Only 150–200 cases are reported in the USA annually [7]. IMT was first discovered in the lungs in 1939 [8], and was considered benign at the time [9]. However, as the understanding of IMT progressed, it was re-classified as an “intermediate” myofibroblastic tumor by the World Health Organization (WHO) due to the discovered chromosomal alteration, nature of local aggressiveness, high recurrence rate, and low metastatic potential [10]. IMT has a recurrence rate of approximately 25%, but it is highly dependent on the IMT location [11, 12]. Studies have reported higher recurrence rates in lesions located in the abdominal space when the tumor size is > 8 cm [6, 13, 14]. In a study evaluating metastatic potential, among 59 documented cases, metastasis was only restricted to six anaplastic lymphoma kinase ALK^−^ IMT cases (10.2%), whereas none of the ALK^+^ IMT cases exhibited metastasis [13]. Younger age, larger tumors, and detection at abdominopelvic and pulmonary sites were indicative of higher metastatic potential [13]. Nevertheless, distant metastasis has only occurred in approximately 5% of cases [12, 15••]. This result was later confirmed by Fu et al., who reported that only five patients with IMT (5.4%) had metastasis among 92 cases [16].

From a clinical perspective, IMT is usually disclosed during routine health checkups [17, 18] because patients can be entirely asymptomatic until the tumor has grown to a size that can cause complications [19]. The presenting symptoms include weight loss and general fatigue [17]. Therefore, the type and severity of symptoms presented by patients with IMT mainly depend on the location of the primary site and size of the tumor [18, 20]. Furthermore, tumor size and patient age are considered prognostic factors; in younger patients or in patients with tumor sizes < 6.5 cm, survival rates tend to be better [16].

**Fig. 1:**
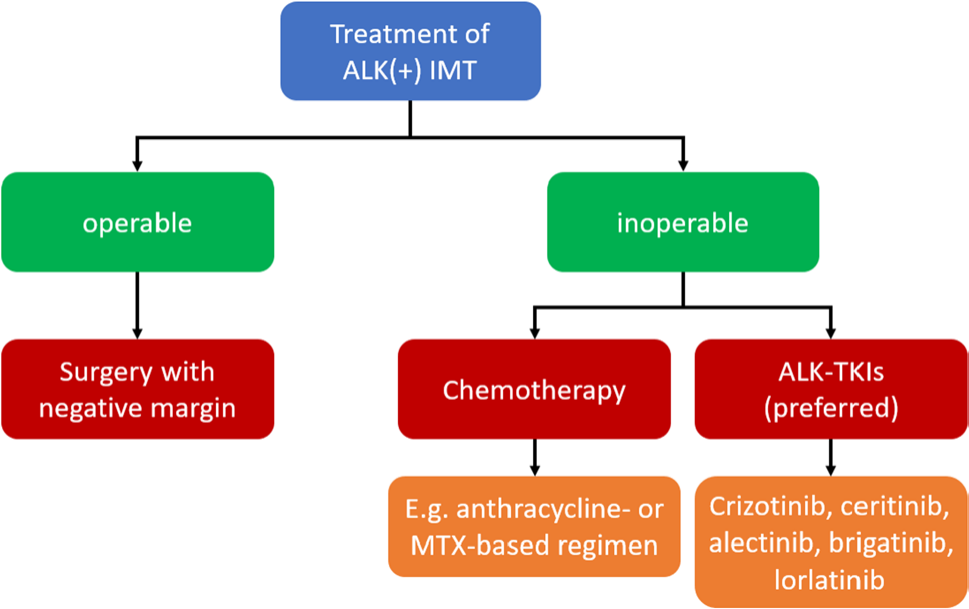
Treatment choices for both operable and inoperable IMT

In most cases, surgical resection is the best approach for IMT treatment, which generally results in a better prognosis [17, 21], especially if the tumor is completely resected with a negative margin [22]. However, if the tumor is inoperable, other treatments should be considered, such as steroids or chemotherapy regimens (e.g., methotrexate, anthracycline-based, or ifosfamide-based) [23–25]. In the 13 of paranasal sinus and nasopharynx IMT cases presented by Zhu et al., better overall survival was correlated with the use of prednisone alone among 10 patients undergoing systemic treatment with a combination of surgery, prednisone, radiotherapy, chemotherapy, or observation alone [26]. However, the number of cases was limited. Therefore, a large cohort study should be conducted to validate this finding. In another study, chemotherapy (anthracycline-based and methotrexate plus/minus vinorelbine/vinblastine regimens) exhibited an objective response rate (ORR) of 47.6% and 53.8% for anthracycline-based or methotrexate-based chemotherapy respectively [27]. The median PFS and OS were 6.3 months and 21.2 months respectively for patients treated with anthracycline-based, and not reached and 83.4 months for methotrexate-based chemotherapy (Fig. 1). No prospective study of chemotherapy in IMT was reported.

Here, we comprehensively reviewed IMT epidemiology and the current methodology for pathological and molecular diagnosis and treatment, particularly for targeted therapy against ALK^+^ IMT.

### Epidemiology

The most common sites of IMT occurrence vary among studies; the lungs [1, 28], abdomen [13, 29], and soft tissues of the limbs or hips [16] have been reported. However, other anatomical sites [29], including the meninges of lobes, spinal cord, orbit, mandible, throat, thorax, heart, liver, duodenum, small intestine, colon, and uterus, have also been reported [16].

In terms of demographics, despite IMT can be diagnosed at any age, it seems to have a predilection for children and young adults [30–32]. Prevalence according to sex has been inconsistent among studies [13, 16, 28, 29]. Overall, the general prevalence of IMT ranges from 0.04 to 0.7%, irrespective of sex or race [33, 34].

Owing to the rarity of IMT, its risk factors are not fully understood. Smoking, minor trauma, and IgG4-related disease are thought to be risk factors for tumorigenesis in IMT [8, 35].

### Pathogenesis

Since it was first described in 1939, the cause of IMT pathogenesis remains unclear. Rohrlich et al. proposed that IMT may be a consequence of cytokine production dysregulation following infection [36]. More investigations had supported this hypothesis, suggesting that IMT may be an unusual immunological response to viruses (such as human herpesvirus 8, and Epstein-Barr virus) [13, 37, 38], surgery, or autoimmune diseases [39]. However, an increasing number of studies have considered IMT to be a tumor rather than a reactive process [33, 40]. The recent discovery of chromosomal abnormalities may indicate that IMT is more of a tumor than an inflammatory result or a pseudotumor [41, 42]. Lovly et al. later confirmed that IMT is a largely oncogene-driven neoplasia [43], and that tumorigenesis is associated with the translocation of receptor tyrosine kinase genes, such as ALK and ROS-1 [22].

### Clinical and pathological features

In the early stages of IMT exploration, a thorough understanding of IMT was difficult to achieve owing to its rarity and similarities with other illnesses, and IMT was commonly confused with inflammatory pseudotumor, fibromyxoid lesions, plasma cell granuloma, or other diseases presenting as inflammatory reactions [1]. Further research on the morphology of IMT, inflammatory spindle cell lesions, revealed a high resemblance to common inflammatory conditions, such as nodular fasciitis and inflammatory fibroid polyps [13, 15••, 31, 44, 45]. Thus, the obstacles mentioned above have made the accurate diagnosis of IMT difficult [3].

**Fig. 2:**
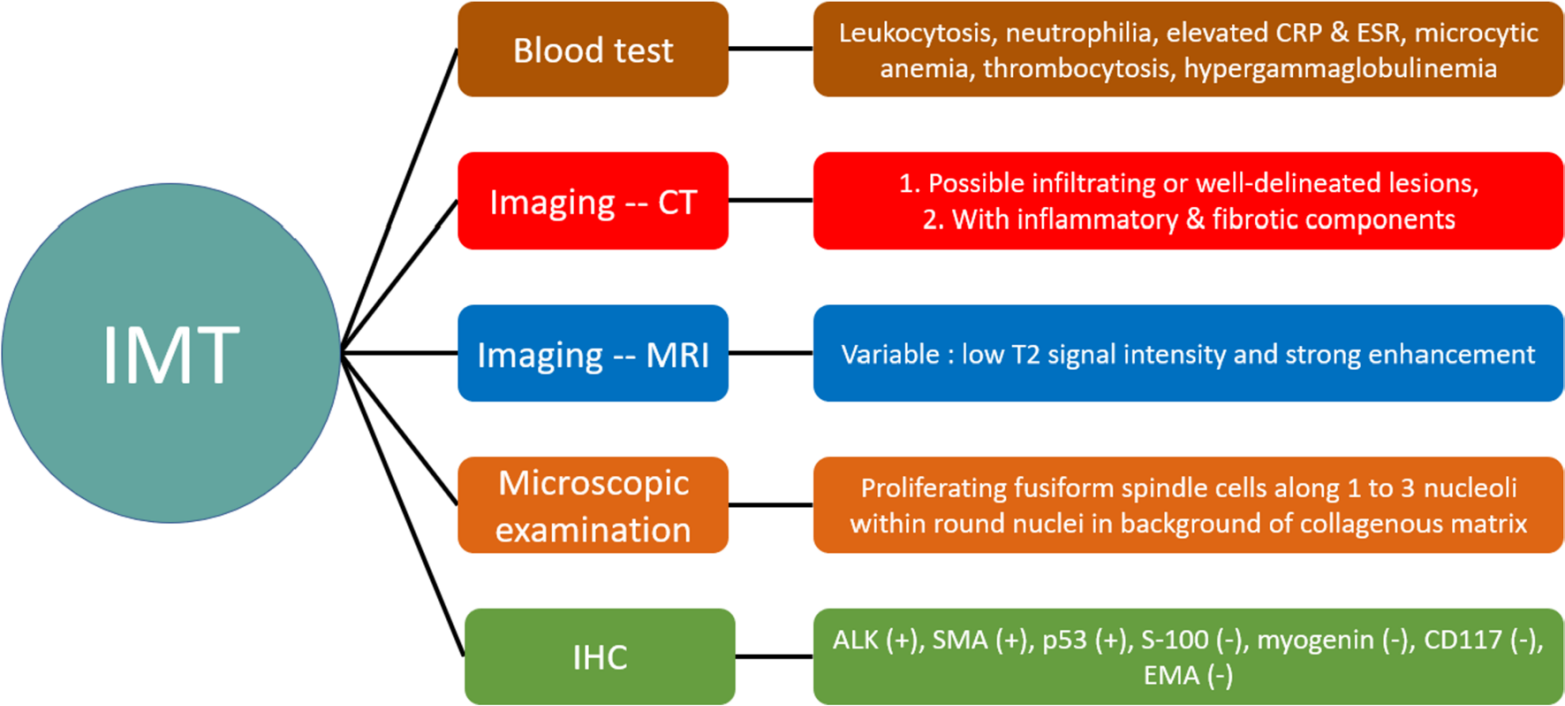
Characteristics of IMT from different aspects

To address this problem, different examination methods have been applied to identify distinctive features of IMT that distinguish it from other similar diseases (Fig. 2). In blood testing, IMT demonstrates leukocytosis, neutrophilia, elevation of C-reactive protein and erythrocyte sedimentation rate [46, 47], microcytic anemia, thrombocytosis, and hypergammaglobulinemia [48]. However, these characteristics are not specific and can be observed in other differential diagnoses, as they are general parameters for inflammation [46]. The radiological morphologies of IMTs located in the soft tissue and bones were similar to those of benign tumors; however, peritumoral edema, parosteal soft tissue, and the invasive rim of IMT were similar to those of malignant tumors [49]. To further resolve this issue, computed tomography (CT) and magnetic resonance imaging (MRI) have been used to differentiate IMT from other neoplasms. Tine-density curves of contrast enhancement by dynamic enhanced CT scanning [50], incidence of calcification, and incidence of the burr sign [51] may help distinguish between peripheral lung cancer and IMT. However, there are various CT/MRI demonstrations of IMTs in other organs, including the mesentery and the musculoskeletal system [52–55]. As seen on CT imaging, IMT morphologies can range from infiltrating lesions to well-delineated lesions with divergent extents of inflammatory and fibrotic components in the mass [20]. MRIs can detect low signal intensity on T1 and T2 weighted images, to reveal IMT fibrosis, with a defined diffusion border [56]. Additional morphological details were discovered using microscopes to aid in the diagnosis. The most identifiable feature is the proliferation of fusiform spindle cells along one to three nucleoli within round nuclei [15] in the collagenous matrix [57], which can be associated with malignant myofibroblasts and dense polymorphic infiltration of mononuclear inflammatory cells [58].

With the increasing importance of differentiating IMT from other spindle cell tumors, IMT has also been examined using immunohistochemistry (IHC), fluorescent in situ hybridization (FISH), and other technologies, such as next-generation sequencing (NGS). The discovery of ALK expression in IMT in 1999 [59] was a breakthrough in IMT diagnosis. It was later found that approximately 50% of the patients with IMT had ALK rearrangements [58]. Furthermore, IMT also presents with wild-type p53 [60], positivity for smooth muscle actin (SMA) [22], and cytokeratin AE1/3 focal positivity. Negative expression of S-100 protein, myogenin, cluster of differentiation 117, and epithelial membrane antigen was reported by IHC [31, 60]. FISH was performed to clarify the reason for ALK overexpression as a result of gene translocation [43, 61]. However, false-negative FISH results can occur for several reasons [61]. This issue can be addressed using NGS. NGS can provide evidence of kinase fusion, and identify the exact fusion partner [43]. Furthermore, NGS has also been proven to be a more reliable method for diagnosing ALK fusion-positive IMT than IHC [22].

In summary, pathological and immunohistochemical tests are considered the gold standard for IMT diagnosis [29]; however, NGS can provide genetic information for more appropriate treatments.

### Genetic alterations in IMT

Following the first identification of ALK in IMT in 1999 [59], Coffin et al. discovered that approximately 50% of patients harbored ALK gene rearrangements [58]. This result was later confirmed by Casanova et al., who reviewed 60 IMT cases, and 40 patients (66.7%) were ALK^+^ [62]. In addition to the discovery of ALK rearrangements, ALK fusion partners have also been identified using NGS [43]. RNA binding protein 2 (RNABP2) [63, 64], insulin-like growth factor binding protein 5 (IGFBP5) [65], tropomyosin 4 (TPM4) [66], sequestosome-1 (SQSTM1) [67], and other fusion partners have been identified [22].

Although ALK rearrangements are significant in IMT studies, other gene rearrangements have also been observed. Antonescu et al. found that 85% of IMTs contain kinase fusions, two-third of which include ALK or ROS proto-oncogene 1 (ROS1)–related fusions [68]. Subsequently, Yamamoto et al. reviewed 40 IMTs diagnosed by FISH and reverse transcription polymerase chain reaction; 72.5% had ALK fusion, 5% harbored ROS1 fusions, 5% displaced neurotropic tyrosine receptor kinase 3, and the rest of the cases were quadruple negative [69]. Given the diagnostic importance of ALK expression in IMT, ALK rearrangement detection has become an approach for differentiating IMT from other conditions [3, 13, 70–74].

These discoveries have been used to predict the prognosis of IMT, and a few studies have suggested that ALK positivity may be an indicator of a better prognosis [11, 13, 37, 75]. In a study by Chun et al., four pediatric patients with IMT underwent incomplete surgical resection; both ALK^+^ patients were successfully treated with follow-up radiotherapy, while those who were ALK^−^ died of the disease [37]. However, the link between fusion partners and the nature of the disease remains unclear [68].

## Current treatment for ALK^+^ non-small-cell lung cancer

Non-small-cell lung cancer (NSCLC) is a general term that describes various morphologies, including adenocarcinoma and squamous cell carcinoma [76]. Moreover, 85% of lung cancers fall into this category [77]. NSCLC usually has a delayed diagnosis because patients are often unaware of the disease and symptoms resemble those of respiratory infections [78]. Thus, among diagnosed NSCLC cases, 40–65% present with distant metastases [79], with an unfavorable 5-year survival rate of merely 5–10% [80].

The treatment of NSCLC depends largely on the stage of the disease, and with delayed diagnosis, surgery is sometimes deemed impossible [76]. In 2007, an ALK rearrangement (EML4-ALK) was identified in NSCLC [81]. Later, abundant research suggested that ALK rearrangements accounted for 5% of cases [82, 83], ushering in a new era in the treatment of NSCLC.

ALK tyrosine kinase inhibitors (TKIs) were then developed as targeted therapies [84]. Crizotinib, a first-generation ALK-TKI, has gained accelerated approval from the US Food and Drug Administration (FDA) for treating either locally aggressive or metastatic NSCLC, based on two single-arm trials, which reported 50% and 61% ORRs [85]. With this success, subsequent generations of ALK-TKIs have also been developed, such as ceritinib, alectinib, brigatinib, and lorlatinib, which demonstrated superior efficacy compared to the first-generation ALK-TKI, crizotinib [86–90].

Due to the shared presence of ALK rearrangements in IMT and NSCLC, comparable effectiveness was anticipated based on tumor-agnostic treatment [84].

## Clinical evidence of ALK-TKIs for ALK^+^ IMT

### Crizotinib

Because IMT and NSCLC share similar ALK expression levels, the efficacy of crizotinib in IMT treatment has been an important area of research for targeted therapy. The first investigation to report satisfactory results with crizotinib in ALK^+^ IMT cases was conducted by Butrynski et al. in 2010 [91]. In the study, two patients with IMT were treated with crizotinib; one with ALK^+^ exhibited a sustained partial response, whereas the other with ALK^-^ exhibited no observable effects [91].

Other investigations have confirmed the efficacy of crizotinib in patients with ALK^+^ IMT. In a time period of 4.5 years, 19 IMT cases were tracked by Schoffski et al., six ALK^+^ patients (50%) and one ALK^−^ patient (14%) displayed an objective response to crizotinib [92]. Based on these results, they proposed that crizotinib may be the standard treatment for patients with locally inoperable, advanced, or metastatic ALK^+^ IMT [92].

In 2022, crizotinib was approved for use in adult and pediatric patients with unresectable, recurrent, or refractory ALK^+^ IMT based on two multicenter, single-arm, open-label trials, including 14 pediatric cases from trial NCT00939770 and seven adult patients from trial NCT01121588 [93••]. The inclusion and exclusion criteria for each study are listed in Supplementary Tables 1 (NCT00939770) and 2 (NCT01121588). In the trial NCT00939770, the ORR was assessed by an independent review committee, and among the 14 cases, 12 patients with IMT (86%) exhibited an objective response [94]. The most common adverse reactions were vomiting, nausea, diarrhea, abdominal pain, rash, cough, pyrexia, fatigue, edema, constipation, and headache [94]. For the trial NCT01121588, an objective response was observed in five (71.4%) of seven patients with IMT, and the most frequent adverse reactions were vision disorders, and edema [95•].

As crizotinib has been approved by the U.S. FDA for the treatment of ALK^+^ IMT, other case reports and case series have not been summarized in this review.

### Ceritinib

Ceritinib, an ALK-TKI, was approved for ALK^+^ metastatic NSCLC with crizotinib intolerance, based on the results of a 2014 trial that included 163 patients [96], and was established as a first-line treatment for ALK^+^ metastatic NSCLC in 2017 based on a phase III trial [97], conducted by the U.S. FDA (recommended dosage = 750 mg orally once daily) [98].

Although it has only been approved for treating ALK^+^ NSCLC, ceritinib has also been reported to be effective against ALK^+^ IMT as an off-label treatment based on the shared characteristics of ALK expression.

Tsakiri et al. reported the case of a 33-year-old man with a TPM4-ALK fusion IMT. Two surgical resections and hyperthermic intraperitoneal chemotherapy were scheduled; however, the tumor relapsed, and crizotinib was chosen as treatment. Although there was an initial response, an activating mutation of p.G1128A in the kinase domain led to the recurrence of IMT and discontinuation of crizotinib, which initiated treatment with ceritinib. Ceritinib (750 mg/day) was prescribed, resulting in 21 months of progression-free survival (PFS) without drug-related toxicity [66]. In another case reported by Trahair et al., a 14-year-old man with an RNABP2-ALK fusion IMT was treated with crizotinib and achieved complete response (CR) as a result. Nevertheless, the patient experienced neutropenia, and the crizotinib dosage was reduced. After the fourth month of crizotinib treatment, widespread recurrence in the abdominal and thoracic spaces was observed, which was countered by increasing the dose of crizotinib to 280 mg/m^2^. The increased dosage stabilized the disease; however, the patient’s condition deteriorated after 1.4 years of treatment [63]. Ceritinib has been used as alternative to crizotinib with CR and PFS consistently maintained for 42 months [63].

Based on the above reports, ceritinib efficacy is similar in treatment of NSCLC and IMT and can overcome crizotinib resistance in IMT caused by previous lines of treatments.

Subsequent reports have confirmed that ceritinib can overcome resistance resulting from ALK mutations after prior ALK-TKI treatment. A 42-year-old women with proline-rich coiled-coil 2B (PRRC2B)-ALK fusion IMT was reported by Wang et al., who was initially treated with crizotinib [99]. Emergence of the ALK R1192P mutation occurred after 5 months of PFS, indicating crizotinib resistance, and the medication was changed to alectinib (600 mg, twice per day), another second-generation ALK-TKI. Alectinib was able to control the disease as partial response (PR) with 5.5 months of PFS. However, the ALK L1196M mutation was detected by NGS which had resulted in disease progression. To resolve this problem, ceritinib treatment was initiated at 450 mg/day. PR was achieved, and PFS lasted for 6 months before switching to lorlatinib [99]. Another case reported by Zhang et al. documented a 22-year-old man with ribosome binding protein 1-ALK fusion IMT who was treated with 250 mg crizotinib twice per day [100]. His condition improved, but full recovery was not achieved. Therefore, alectinib (600 mg twice daily) was prescribed and the patient’s symptoms improved. However, the tumor appeared enlarged on the CT scans. Tumor tissue was collected to identify the underlying cause of the tumor growth. A mutation in ALK L1196Q was observed, but alectinib was continued for another 4 months before substitution with ceritinib. After the initiation of ceritinib (450 mg daily) treatment, PR was observed, and PFS persisted for over 5 months at which time the study was terminated [100].

Additionally, ceritinib has also been used as a first-line treatment against ALK^+^ IMT. In a report by Kyi et al., a 70-year-old women with an IGFBP5-ALK fusion IMT was mis-diagnosed with uterine leiomyosarcoma and treated with pazopanib and multiple lines of chemotherapy in other institutions. After she was transferred to the organization where the authors stayed (Memorial Sloan Kettering Cancer Center, New York, NY, USA), the diagnosis was revised to IMT based on pathological features and ALK expression. After confirmation by FISH and gene fusion detection by MSK-Solid Fusion assay, ceritinib treatment was initiated with PR, and PFS observed for over 24 months. The patient remained on therapy until the study was completed [65].

The detailed characteristics and treatment outcomes of other investigations and studies are summarized in Table 1.

**Table 1:** Ceritinib treatment for ALK^+^ IMT

Authors	Age (year)	Sex	ALK fusion	ALK inhibitor	Line	Response	PFS (months)	Other treatment
Trahair et al. [63]	14.7	M	RANBP2-ALK	Ceritinib	2nd	CR	42+	Crizotinib (1st)
	9.1	M	RNABP2-ALK	Ceritinib	2nd	PR	2	Crizotinib (1st)
Ono et al. [64]	57	M	RANBP2-ALK	Ceritinib	2nd	PR	11	ASP3026 (1st)
Kyi et al. [65]	58	F	FN1-ALK	Ceritinib	2nd	SD	6	Crizotinib (1st)
	68	F	TNS1-ALK	Ceritinib	3rd	PD	2	Crizotinib (1st), alectinib (2nd), lorlatinib (4th)
	61	F	LBH-ALK	Ceritinib	2nd	SD	6	Crizotinib (1st), liposomal doxorubicin (3rd), lorlatinib (4th)
	70	F	IGFBP5 -ALK	Ceritinib	1st	PR	24+	
Tsakiri et al. [66]	33	M	TPM4-ALK	Ceritinib	2nd	CR	21+	Crizotinib (1st)
Wang et al. [99]	42	F	PRRC2B-ALK	Ceritinib	3rd	PR	6	Crizotinib (1st), alectinib (2nd), lorlatinib (4th)
Zhang et al. [100]	22	M	RRBP1-ALK	Ceritinib	3rd	PR	5+	Crizotinib (1st), alectinib (2nd)
Yuan et al. [109]	18	F	NR	Ceritinib	2nd	PR	8	Crizotinib (1st), alectinib (3rd), lorlatinib (4th)
Mittal et al. [120]	11 months	F	NR	Ceritinib	1st	CR	6+	
Brivio et al. [121]	17	M	NR	Ceritinib	1st	CR	26	
			NR	Ceritinib	1st	CR	36+	
	14	M	NR	Ceritinib	1st	70% reduction in size	2	
Michels et al. [122]	36	F	DCTN1-ALK	Ceritinib	2nd	Unconfirmed PR	3.6	Crizotinib (1st)
Mansfield et al. [123]	32	M	TPM3–ALK	Ceritinib	2nd	Significant PR	6+18 (post-surgery)	Crizotinib (1st)

*M* male, *F* female, *PD* progressed disease, *SD* stable disease, *PR* partial response, *CR* complete response, *NR* not reported, *ALK* anaplastic lymphoma kinase, *PRRC2B* proline rich coiled-coil 2B, *FN1* fibronectin 1, *TNS1* tensin 1, *LBH* Limb Bud-Heart, *IGFBP5* insulin like growth factor binding protein 5, *RRBP1* ribosome-binding protein 1, *RNABP2* RNA binding protein 2, *TPM4* tropomyosin 4, *DCTN1* dynactin subunit 1, *TPM3* tropomyosin 3

### Alectinib

In 2017, alectinib was approved by the U.S. FDA for the treatment of ALK^+^ metastatic NSCLC at a recommended dosage of 600 mg twice daily, based on a randomized, multicenter, open-label trial that included 303 patients, ALEX (NCT02075840) [101, 102]. Similar to ceritinib, alectinib is expected to demonstrate equivalent efficacy against ALK^+^ IMT.

In a study conducted by Sunga et al., a 30-year-old woman with a SQSTM1-ALK fusion IMT was successfully treated with surgical resection. However, recurrence occurred 4 months after surgery in the mesentery and omentum, along with the development of a metastatic site in the extraperitoneal space anterior to the bladder. Given the multifocal recurrence, surgical intervention was deemed impossible; thus, the patient was treated with alectinib (600 mg twice per day) owing to a unique ALK translocation. PR was achieved with no novel metastasis, and the PFS duration was > 36 months when the study was completed. The patient experienced fatigue as the only adverse event that had no effect on her livelihood post-treatment [67].

Furthermore, alectinib has also proven its ability to target crizotinib-resistant cases, as described above in the work of Wang et al. in a 42-year-old patient with PRRC2B-ALK fusion IMT [99]. However, it is noteworthy that alectinib may contribute to drug resistance and, therefore, requires supplementation with other ALK-TKIs to achieve an acceptable outcome [99, 100].

The detailed characteristics and treatment outcomes of these and other studies are summarized in Table 2.

**Table 2:** Alectinib treatment for ALK^+^ IMT

Authors	Age (year)	Sex	ALK fusion	ALK inhibitor	Line	Response	PFS (months)	Other treatment
Gros et al. [20]	39	F	RNABP2-ALK	Alectinib	1st	CR	6+	
Rao et al. [61]	21	F	NUMA1-ALK	Alectinib	2nd	Near CR	13+	Crizotinib (1st)
Kyi et al. [65]	68	F	TNS1-ALK	Alectinib	2nd	PR	12	Crizotinib (1st), ceritinib (3rd), lorlatinib (4th)
Sunga et al. [67]	30	F	SQSTM1–ALK	Alectinib	1st	PR	36+	
Wang et al. [99]	42	F	PRRC2B-ALK	Alectinib	2nd	PR	5.5	Crizotinib (1st), ceritinib (3rd), lorlatinib (4th)
Zhang et al. [100]	22	M	RRBP1-ALK	Alectinib	2nd	SD	5	Crizotinib (1st), ceritinib (3rd)
	60	F	TNS1-ALK	Alectinib	2nd	SD	3	Crizotinib (1st)
Yuan et al. [109]	18	F	NR	Alectinib	3rd	NR	8	Crizotinib (1st), ceritinib (2nd), lorlatinib (4th)
Takeyasu et al. [124]	14	M	NR	Alectinib	2nd	CR	44.2	Adriamycin and ifosfamide (1st)
	52	M	CTLC-ALK	Alectinib	2nd	PR	11.5	Adriamycin/ifosfamide (1st), ceritinib (3rd), pazopanib (4th), eribulin (5th)
Saiki et al. [125]	26	M	EML4-ALK	Alectinib	1st	PR	4.4+	
Honda et al. [126]	46	M	SQSTM1–ALK	Alectinib	1st	PR	12+	
Han et al. [127]	56	F	EML4-ALK	Alectinib	1st	PR	16+	
Spafford et al. [128]	29	F	NR	Alectinib	2nd	PD	0	Crizotinib (1st)
Fujiki et al .[129]	6	F	FN1-ALK	Alectinib	1st	PR	2	

*M* male, *F* female, *SD* stable disease, *PR* partial response, *CR* complete response, *NR* not reported, *ALK* anaplastic lymphoma kinase, *PRRC2B* proline rich coiled-coil 2B, *TNS1* tensin 1, *RRBP1* ribosome binding protein 1, *CTLC* Clathrin heavy chain, *EML4* echinoderm microtubule-associated protein-like 4, *SQSTM1* sequestosome 1, *TPM3* tropomyosin 3, *NUMA1* nuclear mitotic apparatus protein 1, *FN1* fibronectin 1, *RNABP2* RNA binding protein 2

### Brigatinib

Brigatinib, another second-generation ALK-TKI, was approved for adult ALK^+^ metastatic NSCLC in 2020 with a recommended dosage of 90 mg daily for the first 7 days, then increased to 180 mg once daily, based on the ALTA 1L (NCT02737501) trial that targeted advanced ALK^+^ NSCLC in adult patients who had not previously received an ALK-TKI [103, 104].

In a report by Xu et al., a 26-year-old man was diagnosed with an RNABP2-ALK fusion IMT, which was successfully treated with crizotinib [105]. However, after 7 months of crizotinib treatment, ascites occurred and an ALK G1269A mutation was detected by Sanger sequencing. To mitigate this, brigatinib (AP26113) was administered daily at a dose of 90 mg. The tumor was 50% smaller after three months of treatment, which qualified as PR. Remission persisted throughout study duration [105].

However, owing to the rarity of IMT and the late approval of brigatinib, larger cohort studies are required to confirm its efficacy in treating ALK^+^ IMT. A clinical trial (Briga-PED, NCT04925609) is in progress to study the efficacy of brigatinib in pediatric and young adult (≤ 25-year-old) patients with ALK^+^ anaplastic large cell lymphoma, IMT, and other solid tumors, with an estimated study completion date by December 2030 [106].

The detailed characteristics and treatment outcomes are summarized in Table 3.

**Table 3:** Brigatinib treatment for ALK^+^ IMT

Authors	Age (year)	Sex	ALK fusion	ALK inhibitor	Line	Response	PFS (months)	Other treatment
Xu et al. [105]	26	M	RNABP2-ALK	Brigatinib	2nd	PR	22.5+	Crizotinib (1st)

*M* male, *PR* partial response, *ALK* anaplastic lymphoma kinase, *RNABP2* RNA binding protein 2

### Lorlatinib

The third-generation ALK-TKI, lorlatinib, was approved for ALK^+^ metastatic NSCLC with a recommended dosage of 100 mg once daily, based on a randomized, multicenter trial, Study B7461006 (NCT03052608) [106]. Although the median PFS was not accessible, an improvement in PFS was observed, and the ORR for the central nervous system was significantly better in the lorlatinib group (82%) than in the crizotinib group (23%) [107].

A 40-year-old man with a TPM4-ALK fusion IMT was reported by Wong et al., and lorlatinib was administered as fourth-line compassionate use therapy. The patient was initially treated with prednisolone without any clinical effects and was enrolled in a clinical trial for treatment with entrectinib, a tropomyosin receptor kinase/ROS1/ALK inhibitor, which delayed disease progression by only three months. In addition, he received a combination of chemotherapy (ifosfamide- and etoposide-based) and radiotherapy for lesions in the brain and chest. Little improvement was observed, and the disease continued to progress with newly formed metastatic sites in the adrenal gland. Thus, lorlatinib was used as the fourth-line treatment, which resulted in PR after 2 months and PFS for 6 months. During lorlatinib treatment, unilateral right-sided lung consolidation was observed, which was suspected to be due to the interaction between infection, radiotherapy, and lorlatinib, requiring treatment with antibiotics and corticosteroids. After 6 months of lorlatinib treatment, the size of the existing brain lesion increased slightly, and the lesion was treated with stereotactic radiotherapy. Brigatinib was administered 3 months after disease exacerbation, ultimately resulting in death [108].

Given the late approval of lorlatinib, major cohort studies are required to verify its efficacy against ALK^+^ IMT. Moreover, although all reported cases of IMT used lorlatinib in later lines of treatment [65, 99, 108, 109], lorlatinib has shown superior efficacy as a first-line treatment treating ALK^+^ NSCLC compared to crizotinib in the CROWN trial (NCT03052608) [110]. Therefore, further studies focusing on lorlatinib as a first-line treatment for ALK^+^ IMT are required.

The detailed characteristics and treatment outcomes are summarized in Table 4.

**Table 4:** Lorlatinib treatment for ALK^+^ IMT

Authors	Age (year)	Sex	ALK fusion	ALK inhibitor	Line	Response	PFS (months)	Other treatment
Kyi et al. [65]	68	F	TNS1-ALK	Lorlatinib	4th	Clinical PD	1	Crizotinib (1st), alectinib (2nd), ceritinib (3rd)
	61	F	LBH-ALK	Lorlatinib	4th	PR	3	Crizotinib (1st), ceritinib (2nd), liposomal doxorubicin (3rd)
Wang et al. [99]	42	F	PRRC2B-ALK	Lorlatinib	4th	SD	5+	Crizotinib (1st), alectinib (2nd), ceritinib (3rd)
Wong et al. [108]	40	M	TPM4-ALK	Lorlatinib	4th	SD	6	Prednisolone (1st), entrectinib (2nd), ifosfamide and etoposide (3rd), brigatinib (5th)
Yuan et al. [109]	18	F	NR	Lorlatinib	4th	CR	42+	Crizotinib (1st), ceritinib (2nd), alectinib (3rd)

*M* male, *F* female, *PD* progressed disease, *SD* stable disease, *CR* complete response, *OS* overall survival, *NR* not reported, *ALK* anaplastic lymphoma kinase, *PRRC2B* proline rich coiled-coil 2B, *TNS1* tensin 1, *LBH* Limb Bud-Heart, *TPM4* tropomyosin 4

## Sequential treatment based on ALK mutation

As the development of ALK-TKIs has become popular in the treatment of different diseases, sequential treatment with these targeted therapies has been tested and may be crucial for maximizing patient survival [111]. Multiple studies have tested different combinations and sequences of TKIs in patients with NSCLC [111–113]. Development of drug resistance after the initial response has a major influence on the sequence of targeted therapies [114]. In a study by Gainor et al., among 103 patients with ALK-rearranged lung cancer, they found that a unique spectrum of ALK mutations may arise for each ALK-TKIs applied, which may result in drug resistance [115]. Moreover, they observed that lorlatinib, a third-generation ALK-TKI, was sensitive to most emerging mutation-related resistances, whereas crizotinib, a first-generation ALK-TKI, was insensitive to most mutations [115].

**Fig. 3:**
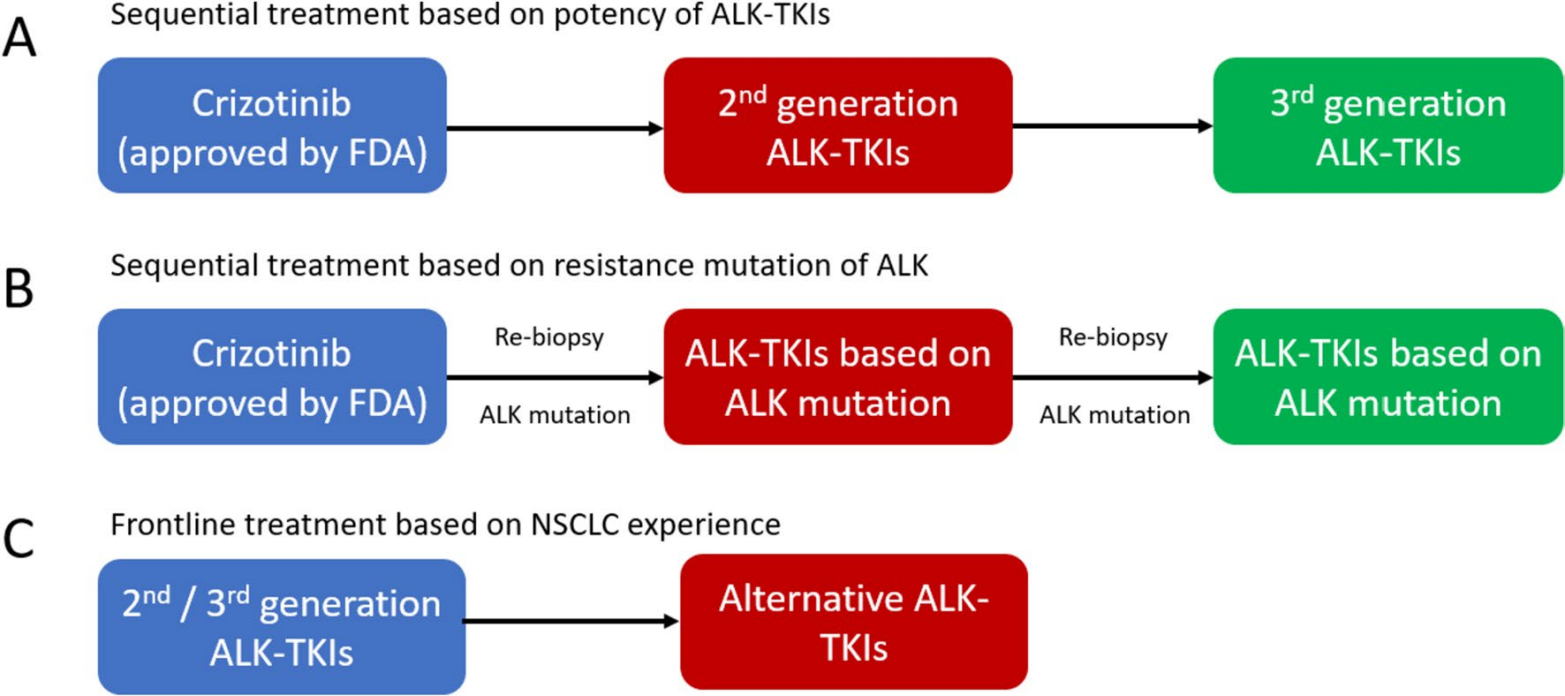
Different strategies of IMT sequential treatments

Given the comparability between ALK^+^ NSCLC and IMT, the treatment sequence for ALK^+^ IMT may require a pattern similar to that used for NSCLC (Fig. 3). Nonetheless, because IMT is a rare neoplasm, data supporting this idea are limited.

Tables 1, 2, 3, and 4 describe the patterns of sequential treatment for different generations of ALK-TKIs (crizotinib, ceritinib, brigatinib, and lorlatinib). Crizotinib is usually used as a first-line treatment, as it has been approved by the FDA [93]; however, the next drug to be used in the sequence remains undetermined. Although ceritinib and alectinib are effective against crizotinib-related resistance, further cohort studies are required to confirm these results. The implicit importance of repeated biopsy and genetic sequencing may direct the next choice of ALK inhibitors as what have been studied in NSCLC.

However, sequential treatment with ALK inhibitors may not be the ultimate solution for ALK-rearranged tumors. Wang et al. reported the case of a 40-year-old man with ALK-rearranged adenocarcinoma. After receiving three different ALK-TKIs (crizotinib, belizatinib, and ceritinib), the patient developed a tumor point mutation under the selective pressure of sequential targeted therapies, resulting in death [116].

## Future challenges

Although some successful treatments using different combinations of ALK-TKIs are reported above, they are mostly presented as “case reports.” Cases with statistically insignificant or negative results were likely to be excluded [117]. Furthermore, confirmation bias may also be a problem. Owing to the success of ALK-TKIs in ALK^+^ NSCLC, IMTs that share similar traits are believed to exhibit compatible results. Hence, there is a tendency to acquire new data in accordance with previous beliefs [118]. To resolve these issues, larger studies are needed.

The diagnosis and treatment of ALK^−^ IMT remain uncertain. Currently, pathological features and IHC tests are viewed as standard procedures to confirm the presence of IMT [29]; however, those with ALK^-^ expression are still difficult to identify, and can be easily confused with similar diseases, such as pseudotumors [22]. In 2022, Zhu et al. reported their experience in treating eight patients with pulmonary IMT, and proposed that vimentin and SMA may be important markers for diagnosing IMT [119]. The accuracy of this result needs to be tested in larger studies, but it still brings hope for IMT diagnosis, even with negative ALK expression. Given the absence of ALK expression, ALK-TKIs are not as useful as in ALK^+^ IMT treatment. Although surgeries with negative margins are still considered the best approach, treatment for inoperable ALK^-^ IMT may remain with traditional measures for neoplasms, such as chemotherapy [62].

## Summary

IMT has nature of local aggressiveness, high recurrence rate, and low metastatic potential. Surgical resection is still the main therapeutic method for localized IMT. Once IMT develops to locally advanced (unresectable) or metastatic, systemic treatment should be applied. Anthracyclin- or methotrexate-based regimens are the potential options even lacking of prospective studies.

After the approval of crizotinib targeting ALK^+^ IMT, ceritinib and other generations of ALK-TKIs have proven their efficacy in some cases, and follow a similar pattern as in ALK^+^ NSCLC, which should be further confirmed with cohort studies. Moreover, the efficacy of lorlatinib as a first-line treatment should also be tested, given its success in ALK^+^ NSCLC. A basket trial is suggested to verify whether the efficacy of ALK-TKIs is optimal, and whether other factors have any impact on the drugs. Sequential treatment for ALK^+^ IMT based on mutation-related drug resistance remains to be developed.

Many questions regarding IMT remain to be answered. Although it is a rare neoplasm with a low recurrence rate, any groundbreaking advancement could be advantageous when faced with other diseases in similar contexts.

### Supplementary information

ESM 1 (DOCX 27 kb)
