# Supplementary material for: Update of Diagnosis and Targeted Therapy for ALK+ Inflammation Myofibroblastic Tumor
Source: Curr Treat Options Oncol. 2023 Nov 8;24(12):1683–702. doi: 10.1007/s11864-023-01144-6 (PMC10781869; doi:10.1007/s11864-023-01144-6)
Supplement: Supplementary file 1 — (DOCX 27 kb) [file 11864_2023_1144_MOESM1_ESM.docx]

| **Supplementary Table 1. Inclusion and exclusion criteria for NCT00939770** | |
| --- | --- |
| **Inclusion criteria** | **Exclusion criteria** |
| - Body surface area $\geq0.63 m^{2}$ at the time of study enrollment. - Histological verification of malignancy at the original diagnosis or relapse. - Patients with relapsed or refractory solid tumors or ALCL (excluding patients with primary or metastatic central nervous system tumors or patients with primary cutaneous ALCL). (Phase 1, Part A1) - Patients with confirmed ALK fusion proteins, ALK mutations, ALK amplification (defined as greater than four-fold increase in the ALK signal number as compared to reference signal number on chromosome 2q arm); ALK IHC can be used as a surrogate for FISH for patients with IMT or ALCL. - Patients must have either measurable and/or evaluable disease. - Performance level   - Karnofsky $\geq$ 50 for patients $>$ 16-year-old   - Lansky $\geq$ 50 for patients $\leq$ 16-year-old - Must have fully recovered from the acute toxic effects of all prior anti-cancer therapies - Patients with solid tumors must not have received chemotherapy within three weeks of enrollment in this study (six weeks if prior nitrosourea). - At least seven days since the completion of therapy with a growth factor. - At least seven days since the completion of therapy with a biologic agent, the duration of this interval must be discussed with the study chair. - At least seven days or three half-lives, whichever is longer, must have elapsed since prior treatment with a monoclonal antibody. - At least 42 days after the completion of any type of immunotherapy, e.g. tumor vaccines. - Patients must not have received prior crizotinib therapy. - Bilirubin $\leq$ 1.5 times of upper limit of normal for age. - Serum glutamate pyruvate transaminase $\leq$ 110 U/L - Serum albumin $\geq$ 2 g/dL - Corrected QT interval (QTc) $\leq$ 480 msec - Patients taking the capsule formulation must be able to swallow capsules; feeding tube administration was allowed for patients receiving an oral solution. | - Pregnant or breast-feeding women were not included in this study; man or women of reproductive potential could not participate unless they agreed to use an effective contraceptive method. - Patients receiving corticosteroids who have not been on a stable or decreasing dose of corticosteroid for the prior seven days were not eligible. - Patients who were currently receiving other investigational drugs were not eligible. - Patients who were receiving other anti-cancer agents, with the exception of hydroxyurea for patients with ALCL, were not eligible. - As crizotinib is an inhibitor of cytochrome P450, family 3, subfamily A, polypeptide 4 (CYP3A4), patients chronically receiving medications known to be metabolized by CYP3A4 and with narrow therapeutic indices including pimozide, aripiprazole, triazolam, ergotamine and halofantrine were not eligible; the topical use of these medications (if applicable) was allowed. - Patients chronically receiving drugs that are known potent CYP3A4 inhibitors within seven days prior to study enrollment, including but not limited to, ketoconazole, itraconazole, miconazole, etc., were not eligible; the topical use of these medications (if applicable), e.g. 2% ketoconazole cream, was allowed. - Patients chronically receiving drugs that are known potent CYP3A4 inducers within 12 days prior to study enrollment, including but not limited to carbamazepine, phenobarbital, etc., were not eligible; the topical use of these medications (if applicable) was allowed. - Patients with known interstitial fibrosis or interstitial lung disease were excluded. - Patients with a known history of myocardial infarction or cerebrovascular accident were excluded. - Patients with CNS tumors or known CNS metastases were excluded; patients with a history of surgically resected CNS metastases were eligible only if the baseline evaluation showed no evidence of current CNS metastases - Patients with uncontrolled infections were excluded. - Patients who in the opinion of the investigator may not be able to comply with the safety monitoring requirements of the study were excluded. |
| The criteria above are a summary of the terms related to IMT. Please refer to the ClinicalTrial.gov for further details. | |

| **Supplementary Table 2. Inclusion and exclusion criteria for NCT01121588** | |
| --- | --- |
| **Inclusion criteria** | **Exclusion criteria** |
| - Histologically or cytologically proven diagnosis of malignancy other than NSCLC. - Positive for translocation or inversion event involving the ALK gene locus. - Positive for ALK amplification events. - Positive for ALK activating point mutations. | - Amplification mutations involving the c-Met gene but not the ALK gene. - Concurrent treatment in another therapeutic clinical trial. - Prior therapy specifically directed against ALK. |
| The criteria above are the summary of terms related to IMT. Please refer to ClinicalTrial.gov for further details. | |
